# Radiation-Grafted Polymer Electrolyte Membranes for Anhydrous Fuel Cell Operation

**DOI:** 10.1021/acspolymersau.5c00142

**Published:** 2026-01-21

**Authors:** Kevin R. Mecadon, Zois Tsinas, Joseph W. F. Robertson, Markus Bleuel, Eric D. Wachsman, Fred B. Bateman, Mohamad I. Al-Sheikhly

**Affiliations:** † Department of Materials Science and Engineering, 1068 University of Maryland, College Park, Maryland 20742, United States; ‡ Theiss Research, La Jolla, California 92037, United States; § Materials Measurement Science Division, 10833 National Institute of Standards and Technology, Gaithersburg, Maryland 20899, United States; ∥ Microsystems and Nanotechnology Division, National Institute of Standards and Technology, Gaithersburg, Maryland 20899, United States; ⊥ NIST Center for Neutron Research, National Institute of Standards and Technology, Gaithersburg, Maryland 20899, United States; # Radiation Physics Division, National Institute of Standards and Technology, Gaithersburg, Maryland 20899, United States

**Keywords:** polymer electrolyte membrane, fuel cell, heterocyclic amine monomers, electrochemical impedance spectroscopy, proton conductivity, electron beam

## Abstract

Herein, we describe the design, synthesis, and analysis of anhydrous fuel cell membranes that can operate at temperatures above 100 °C, in view of enhanced performance and stability. Traditional polymer electrolyte membrane fuel cells (PEMFCs) do not operate efficiently above 100 °C because water is used as a proton-conductive medium through the Grotthuss hopping mechanism. By substitution of water with heterocyclic amine monomers and use of ionizing radiation to graft them onto fluoropolymer films, proton-conductive network solid-state polymer electrolyte membranes (PEMs) were developed. PEMs were synthesized using indirect radiation grafting of the following heterocyclic amine monomers: 4-vinylpyridine and 5-vinylpyrimidine onto fluorocarbon substrates. The resulting PEMs have proton conductivities greater than 10^–2^ S/cm above 100 °C and perform independent of humidity conditions. These PEMs also demonstrate a positive correlation of increased proton conductivity with increasing temperatures above 100 °C. The chemical properties and structures of the grafted monomers affect the proton-conductive mechanism and performance of the PEMs. The data generated through this research will further the development of anhydrous PEMs through radiation grafting to achieve higher proton conductivity, enhanced performance, and stability.

## Graphical abstract

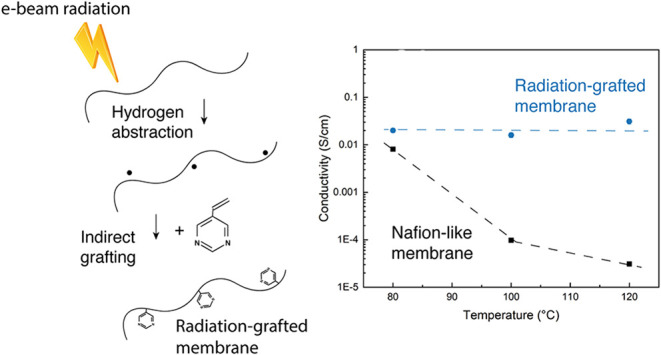

## 1 Introduction

Higher temperatures enable fuel cells to operate more efficiently by enhancing reaction kinetics, increasing catalytic activity, and decreasing carbon monoxide poisoning of the electrodes.^1^ However, to operate at higher temperatures, water needs to be replaced as the proton-conducting medium; otherwise, the PEMs would dehydrate and lose proton conductivity. Current PEMFCs have limits to their operating window and have extensive support systems to maintain the temperature and humidity within the fuel cell. Heterocyclic amine and acidic functional groups have been explored as alternative proton-conductive media to produce high-temperature PEMs. Heterocyclic amine-containing membranes exhibit high proton conductivity at high temperatures and can compete with current PEMs that use water for proton conductivity.^2^ Radiation grafting of copolymer membranes has become an alternative for the design and development of new PEMs. Radiation-induced graft polymerization of styrene produces styrene sulfonic acid PEMs with good proton conductivity but poor mechanical properties.^3,4^ Sulfuric and phosphoric acid treatment of membranes is another chemical method that promotes hydrogen bonding within PEMs to improve water retention and proton conductivity at elevated temperatures.^5^ These acidic functional groups are incorporated with high-temperature acid treatments that can damage the PEM durability and stability. To improve the reliability of acid-treated PEMs and to prevent their degradation, a radiation grafting procedure was studied to chemically bond proton-conductive functional groups directly onto fluorocarbon films, which have high electrochemical resistance.^6^

Another area of PEM research is the incorporation of ionic liquids and other heterolytic amine monomers onto porous polymer membranes as a means of proton conductivity via grafting, cross-linking, or other copolymerization techniques, in order to create anhydrous, high-temperature, solid-state systems.^7–10^ Ionic liquids substituted for water as the proton-conductive medium have a positive correlation with the proton conductivity and temperature. Proton conductivity of ionic liquids improves as their number of charge carriers increases, their viscosity decreases, and when the material overall has lower conjugation.^11^ Polymers that have been used to create polymeric ionic liquids, such as poly-4-vinylpyridine, poly-2-vinylpyridine, and poly­(vinylimidazoline), have been successfully acid-treated to generate PEMs.^9,10,12^ Furthermore, ionic liquids have been incorporated into Nafion[Fn fn1] and fluorocarbons to improve the proton conductivity. Free radical polymerization was previously used to covalently radiation-graft ionic liquids onto PEMs.^13^ Finally, imidazole ionic liquids were covalently bonded to the polymer substrate to prevent evaporation and loss of proton conductivity.^14^

In this work, we used radiation-induced graft polymerization to attach heterocyclic amine monomers into polymer membranes to develop an anhydrous proton-conductive network. The fluorocarbon polymers serve as the backbone of the PEM and were selected based on their properties to withstand the oxidation/reduction reactions and high temperature for fuel cell operation. Fluorinated ethylene-*co*-propylene (FEP), poly­(vinyl fluoride) (PVF), and poly­(chlorotrifluoroethylene) (PCTFE) were chosen. These fluorocarbon polymers are chemically resistant with high melting points and low electrical conductivity.^15^ In addition, FEP, PVF, and PCTFE were selected for their chemical structure to provide a high degree of radiation resistance and reduce backbone scission during the synthesis process.^16,17^ If backbone scission occurs, the average molecular weight of the polymer is reduced, decreasing the mechanical properties, long-term stability, and performance of the PEM. For proton conductivity, heterocyclic amine monomers 4-vinylpyridine and 5-vinylpyrimidine were chosen in this study since their structures are symmetric and have vinyl groups suitable for radiation grafting onto the fluorocarbon substrates to create PEMs. The monomer symmetry decreases the activation energy for proton conductivity between grafted monomer groups. In addition, the use of ionic liquids like monomers has several advantages and is characterized by high ionic and proton conductivity, low vapor pressure, high electrochemical stability, high thermal stability, and high decomposition temperatures.^18^ Of the three types of ionic liquids: aprotic, protic, and zwitterionic, only protic ionic liquids are suitable for solid-state proton conductivity. Protic ionic liquids, similar to water, are able to exchange protons with neighboring groups. The objectives for this research were to design, synthesize (utilizing radiation grafting techniques), and analyze innovative anhydrous PEMFC membranes that operate at temperatures above 100 °C. These new PEM materials were designed to operate with a different proton transport mechanism, which maintains proton conductivity at high temperatures. The inter-relationship between the chemical/physical properties and proton conductivity of the membrane was studied to help the development of future anhydrous fuel cell membranes.

## 2 Experimental Section

### 2.1 Materials

Fluorinated ethylene-*co*-propylene (FEP), poly­(vinyl fluoride) (PVF), and poly­(chlorotrifluoroethylene) (PCTFE) were provided by CS Hyde, Goodfellow, and Honeywell, respectively. The thickness of these membranes was 25 μm. Heterocyclic amine monomers 4-vinylpyridine were from Sigma-Aldrich and 5-vinylpyrimidine from Ryan Scientific Inc.

### 2.2 Indirect Radiation Grafting

PEMs were synthesized at the Medical Industrial Radiation Facility (MIRF) at the National Institute of Standards and Technology (NIST). A nominally 10.5 MeV electron beam accelerator with a dose rate of 1000 kGy/h was used to indirectly radiation-graft the heterocyclic amine monomers onto fluorocarbon polymer substrates to create PEMs. Each sample was irradiated at total absorbed doses of 25, 50, or 100 kGy. The delivered dose of the electron beam was calibrated via thin-film alanine dosimetry. Irradiations were performed under a dry ice environment at about −78.5 °C. The vials containing the membranes were purged with argon and nitrogen before the irradiation process to ensure that no oxygen was present. Immediately after irradiation of the substrates, the monomers were added in an inert argon environment and placed in an oven for post-heat treatment (PHT) at 80 °C for 5 h to ensure uniform grafting. Then, the grafted membranes were washed via sonication in methanol and then ethanol to remove any ungrafted material. Membranes were dried and weighed; this process was repeated until a stable mass was obtained. Further details about the selection of the appropriate irradiation conditions for the samples can be found in Section 3.1.

### 2.3 Degree of Grafting

The synthesized PEMs were analyzed to evaluate the amount of monomer grafted onto the substrate. Percent grafting was determined using the gravimetric formula, eq 1.

1 $$\mathrm{degree}\;\mathrm{of}\;\mathrm{grafting}=\frac{M_{f}-M_{i}}{M_{i}}\times 100$$

The initial mass (*M*_i_) of fluorocarbon substrates and the final mass (*M*_f_) of the PEM were used to determine the degree of grafting from the radiation synthesis. The sample mass of substrate and PEM materials was between 100 and 300 mg.

### 2.4 Free Radical Determination

Electron-paramagnetic resonance (EPR) spectroscopy was used to study the formation of unpaired electron centers in the radiation-treated fluoropolymer substrates. Because the free radicals are potential grafting sites, their characterization is important to understand the quality of the grafting. Monitoring the EPR spectra over time can provide valuable information on the number of radicals available for grafting, which is important, especially for indirect radiation grafting, where there is a time delay between radiation treatment and monomer addition. Room temperature (25 °C) EPR spectra were collected at NIST on a commercial, continuous wave (CW) spectrometer operating at X-band (9 to 10 GHz). Spectra were collected with the following parameters: microwave frequency = 9.822 GHz, microwave power = 0.6362 mW, modulation frequency = 100 kHz, modulation amplitude = 0.5 mT, receiver gain = 50, center field = 348.0 mT, sweep width = 30 mT, conversion time = 40.96 ms, and time constant = 20.48 ms. The doubly integrated areas of the EPR signals were used to monitor changes in the concentration of free radicals in the samples over time.

### 2.5 Morphological and Elemental Composition Analysis

The surface morphology and cross-section of the PEMs were characterized by scanning electron microscopy (SEM) using a variable-pressure SEM instrument equipped with an X-ray detector that allows for elemental analysis through energy-dispersive spectroscopy (EDS). Membranes were prepared by embedding them in epoxy, cross-sectioning, and finely polishing. The samples were then gold (Au) sputter-coated (coating thickness was about 5 nm), and analysis was conducted under high vacuum at 8 keV. The SEM/EDS analysis was used to determine the uniformity of PEM density and relative composition upon grafting. EDS composition percentages were normalized to carbon to better identify the changes in amine groups present on the substrates prior to and post grafting.

The synthesis of grafted PEMs was also confirmed by using Fourier Transform Infrared (FTIR) spectroscopy. Attenuated total reflectance (ATR)-FTIR spectra before and after radiation grafting were collected to show the chemical changes that occur during grafting. Additional spectra were collected after the grafted samples were treated with various acids to protonate the PEMs. The spectra presented in this work are an average of 124 scans at a resolution of 4 cm^–1^. Small pieces of the samples were secured between the ATR crystal and the push-down pin of the instrument at a constant pressure to ensure maximum contact of the sample with the crystal window.

### 2.6 Conductivity Evaluation

Electrochemical impedance spectroscopy (EIS) measurements were performed in-plane via a 4-point test cell to determine the proton conductivity of the developed PEMs. These measurements were performed by using a commercially available potentiostat (Solartron Modulab ECS). The samples were placed inside an environmental chamber with humidity and temperature control. Prior to testing, PEMs were cut into strips and boiled in deionized water for 30 min to remove residual ions. Then, membranes were protonated by an acid treatment using an aqueous acidic solution at a volume fraction of 0.05 of either HNO_3_, H_2_SO_4_, or H_3_PO_4_ at 50 °C for 1 h. The membranes were double rinsed with deionized water to remove residual acid. Proton conductivity was measured by applying an alternating current (AC) signal, either voltage (10 mV) potentiostatic or current (1 μA) galvanostatic, at varying frequencies, and then measuring the system response. For PEMs, a phase shift between the AC signal and response was expected due to double-layer capacitance and redox reaction kinetics of proton transport at the electrodes. Measurements were collected across the frequency range of nominally 10^6^ Hz to 1 Hz. Data were analyzed by fitting to equivalent circuit models, and the observed time constants were attributed to mechanisms using distribution of relaxation time (DRT) analysis.^19^ In each case, error estimates were derived from the standard deviations of three independent measurements.

### 2.7 Microstructure Determination

Small-angle neutron Scattering (SANS) measurements were used to investigate changes in the internal morphology of the membranes before and after grafting of the monomers. SANS measurements were conducted on the 30 m NG7 SANS instrument at the NIST Center for Neutron Research (NCNR).^20^ Samples were prepared by folding PEM membranes into 10 layers of approximately 1 cm^2^ surface area and were compressed between quartz windows by an aluminum sample holder. Sample-to-detector distances of 1, 4, and 13 m were collected with a nominal neutron wavelength of 0.6 nm (6 Å), and an additional setting at 13 m using a neutron lens, with 0.8 nm (8 Å) wavelength neutrons, was used resulting in a scattering range of 0.01 nm ^–1^ < *q* < 0.78 nm ^–1^, and reduced with NCNR data reduction macros in Igor Pro 8 (Wavemetrics, Portland, OR).^21^ Data were fit using SASView5 (http://www.sasview.org/). Error analysis was performed using the DREAM fitting algorithm, an adaptive Markov Chain Monte Carlo simulation^22^ with 10,000 samples and 1000 burn-in steps to avoid slowly converging fits.

## 3 Results and Discussion

### 3.1 Indirect Radiation Synthesis

Fluorocarbon substrates were exposed to a high-energy ionizing radiation source at the NIST (electron energies of 10.5 MeV) to generate free radicals on the polymer backbone. During radiation treatment, dry ice was used to cool samples to −78.5 °C to reduce the mobility of generated radicals, decrease cross-linking reactions, and preserve them for grafting of the desired monomers. The radiation-induced free radicals react with the double bond of the selected monomers to graft directly onto the fluorocarbon substrate to produce PEM membranes. A post-heat treatment temperature of 80 °C, upon addition of the monomers, was selected to be above the glass transition temperature of the fluorocarbon substrates to allow uniform diffusion and bulk grafting. However, the higher the temperature, the greater the radical mobility and the probability of undesired cross-linking. These competing reactions from the indirect radiation grafting method are diagrammed in Figure 1. During radiation treatment, undesired side reactions, including backbone chain scissions and interchain cross-linking, as shown in Figure 1 reaction (c), may also occur. Optimization of the irradiation parameters was conducted to promote radiation-induced grafting reactions (reactions (a) and (b) shown in Figure 1) over undesired reactions. Similar competing reactions are possible for PCTFE and PVF during the radiation treatment.

**Figure 1:**
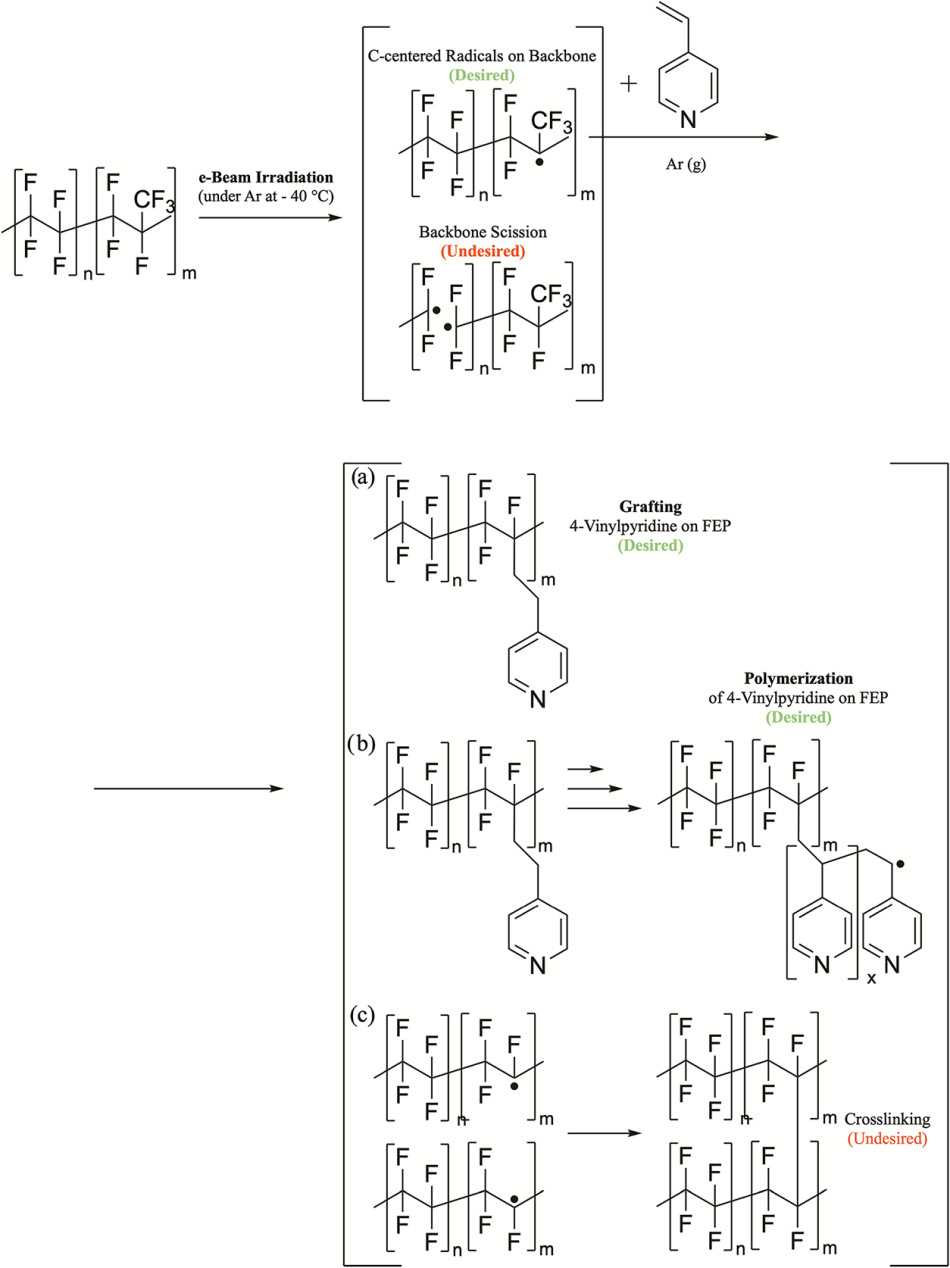
Desired and undesired reactions of indirect radiation grafting synthesis of FEP-polymer electrolyte membranes.

Following sample irradiation, radicals are formed along the backbone of the polymer through either defluorination or backbone chain scissions.^16,23^ Carbon-centered free radicals in fluoropolymers have longer lifetimes than in hydrocarbon polymers due to their lower mobility along the chains, as determined by longer half-lives in EPR spectroscopic measurements.^24^ The longer half-life is due to the greater steric hindrance of fluorine versus hydrogen in the backbone. The electronegativity of the fluoride groups reduces the mobility of electrons along the backbone. By testing a range of irradiation parameters, it was determined that high-dose rates produce higher and more uniform grafted PEMs. However, the dose levels should be optimized for each specific monomer and polymer substrate combination to achieve bulk radiation grafting and create a uniform structure. The degree of grafting is controlled by the radiation dose, dose rate, and temperature. The use of radiation-induced grafting for membrane fabrication has been successful in a wide range of applications.^25^ Because ionizing radiation penetrates the entire thickness of the substrate material, fuel cell membranes can be fabricated in such a way that the ion-conducting monomer is deeply and evenly embedded within the substrate polymer. After samples are irradiated and cooled, the indirect grafting synthesis was performed under an inert atmosphere by bubbling the samples with Argon while adding the desired monomer. The Argon purge was performed to minimize oxygen from reacting with the free radicals generated in the fluorocarbon substrate. A post-heat treatment was performed at a temperature above the glass transition temperature of the grafted polymers for 5 h at 80 °C to allow uniform monomer diffusion and bulk grafting according to the grafting front model, as sketched in Figure 2.^26^

**Figure 2:**
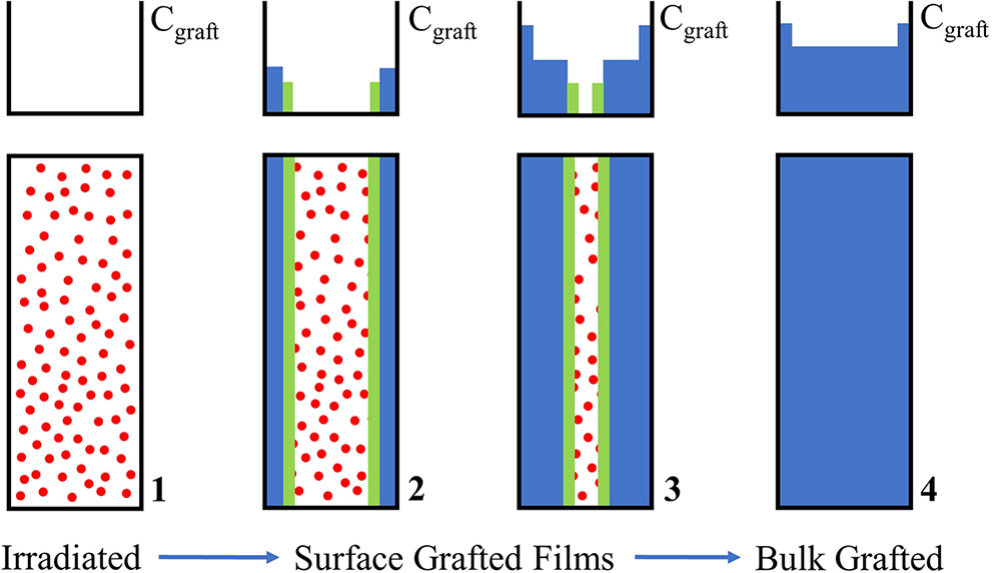
Grafting Front Model: PEM synthesis; the sketch shows step by step the process of monomer diffusion (green) and grafting (blue) onto the C-centered radicals (red dots) of the substrate membrane (cross-section concept sketches—side view).

During the first step, (1) radiation generates free radical active sites for grafting, which are represented as red dots. Then, (2) grafting is initiated at the surface by polymerization of monomers from solution. (3) Further grafting of active sites within the irradiated film requires diffusion of monomers through the already grafted polymer zone. Over time, the grafting front, depicted in green, moves from the surface to the center of the membrane. Further grafting increases the concentration of the monomer in the membrane. Finally, (4) grafting for an extended time yields homogeneous grafted films with a uniform concentration over the entire film thickness. Since ionizing radiation penetrates the entire depth of the substrate material, fuel cell membranes can be fabricated in such a way that the ion-conducting monomer is evenly embedded within the substrate polymer. Uniformly grafted samples were prepared using a high radiation dose rate of 1000 kGy/h (2.31 Gy/pulse) at a radiation temperature below −40 °C. Both 4-vinylpyridine and 5-vinylpyrimidine were grafted to the fluorocarbon substrates (FEP, PCTFE, and PVF) under inert conditions, a dry ice environment, and post-heat treatment, upon the addition of the monomers, at nominally 80 °C for 5 h. The dose rate, dose, and average percent grafting values of each sample discussed in this work are presented in Table 1

**Table 1:** Percent Grafting of 4-Vinylpyridine and 5-Vinylpyrimidine onto Fluorocarbon Substrates via High-Dose Indirect Radiation Grafting^a^

sample	polymer substrates	heterocyclic amine monomer	dose (kGy)	average grafting (%)
I	FEP	5-vinylpyrimidine	25	19.6 ± 1.4
II	PCTFE	5-vinylpyrimidine	100	11.8 ± 1.2
III	PVF	5-vinylpyrimidine	25	44.6 ± 2.2
IV	FEP	4-vinylpyridine	50	18.6 ± 2.0

a The data represented are mean ± standard error of the mean (S.E.M.), where *n* = 5.

During the irradiation experiments conducted to synthesize PEMs, parameters such as the dose rate and total dose delivered were optimized to achieve the highest percent grafting for each material. The results show that a dose rate of 1000 kGy/h can lead to average grafting values of 10% by weight or higher. However, not all of the synthesized PEMs had achievable grafting values above 10% by weight. These PEMs had inadequate performance and are not presented in this work. Also, the results show that there was no direct correlation between total irradiation dose and grafting density for the selected materials. Optimization of the delivered total dose was necessary for each formulation to achieve the highest possible average grafting values. The difference in the grafting behavior trends is likely due to the mobility of free radicals in the different fluorocarbon substrates and the competing reactions between grafting and cross-linking, as well as the radiation resistance properties of each substrate.

### 3.2 EPR Analysis of Free Radicals in Fluorocarbon Substrates

The presence of free radicals within the irradiated fluorocarbon substrates, FEP, PCTFE, and PVF, was determined by electron-paramagnetic resonance (EPR) spectroscopy. The time-dependent production of free radicals from radiation treatment is important to allow for grafting of the monomers onto the fluorocarbon substrates, especially when indirect radiation grafting is implemented. The EPR spectra for irradiated fluorocarbon substrates were collected at room temperature and are displayed in Figure 3(A). For this set of experiments, all substrates were irradiated under the same conditions with a total dose of 50 kGy and a dose rate of 1000 kGy/h. The peaks in the EPR spectra represent free radicals present within each sample immediately after irradiation. Although some of the materials produced complex EPR spectra postirradiation, we did not attempt to deconvolve or fit the signals in the spectra. Instead, we monitored the decays of the EPR signals to ensure that sufficient radical amounts were present for grafting, as shown in Figure 3(B–D). Monitoring the EPR spectra over time establishes the maximum allowable time between radiation treatment and monomer addition for the indirect radiation grafting method we used (Figure 3).

**Figure 3:**
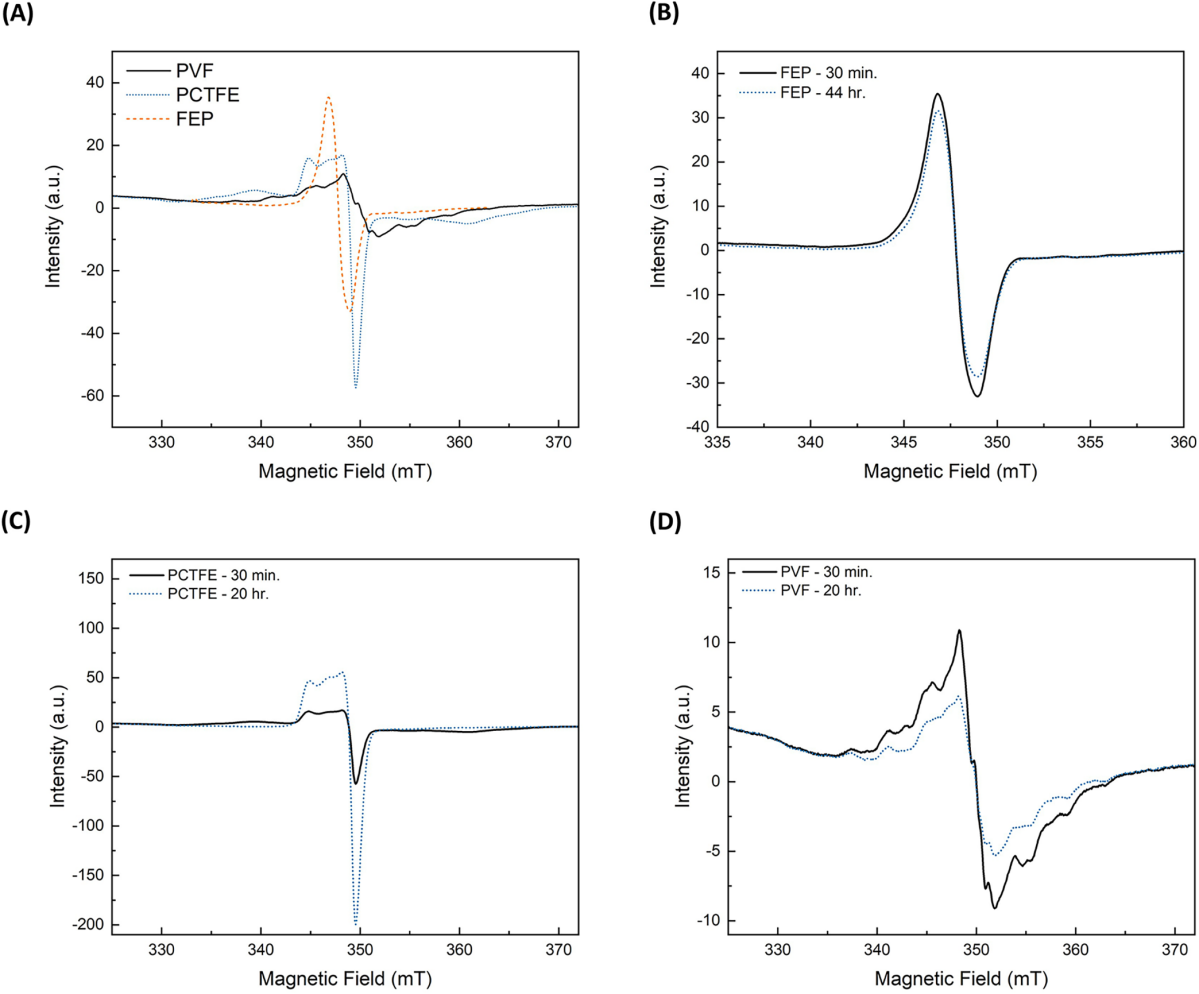
EPR spectra of irradiated substrates at a dose rate of 1000 kGy/h and a total absorbed dose of 50 kGy; (A) continuous wave EPR spectra of irradiated PVF, PCTFE, and FEP substrates 30 min postirradiation; and (B–D) EPR signal decay of irradiated polymer substrates after 20 h stored at ambient temperature and in the absence of oxygen.

For FEP, a single feature was observed with an approximate *g*-factor of 2.018. Because FEP contains both C–F and C–CF_3_ bonds, ionizing radiation produces homogeneous bond cleavage at both sites; however, the highly electron-withdrawing trifluoromethyl group weakens the C–CF_3_ bond, making it four times more likely to break after radiation treatment compared to the C–F bond. A prior study by Gehringer et al. on radiation of perfluoromethylcyclohexane calculated radiation-chemical yield g-factors, which indicated that it was more probable for the fluorinated methyl group bonds to break rather than the hexane ring to open.^27^ This finding potentially explains the observed greater radiation resistance of FEP over poly­(tetrafluoroethylene) (PTFE) because backbone scissions are less likely to occur. As for the PCTFE and PVF, the EPR spectra, shown in Figure 3(A), are more complex with partially resolved hyperfine structure and/or contributions from multiple radical species, one of which is likely a peroxyl radical formed from the reaction of the radiation-induced radical on the polymer with oxygen.

According to the data shown in Figure 3(B–D) and in Table 2, the free radicals decayed slowly in all samples, so that sufficient amounts of radicals are available for indirect grafting after a hold time of 15 min post radiation treatment. Table 2 shows that the decay of free radicals over the time course we measured (*t* ≥ 20 h) is inversely proportional to the degree of fluorination FEP > PCTFE > PVF. FEP, having the highest degree of fluorination, showed a nominal 10% decrease in the total area that correlates to carbon-centered radicals even after 40 h, compared to PVF, which has the lowest degree of fluorination and revealed a nominal 39% decrease after 20 h. This result is expected based on previous literature, indicating that fluoride groups immobilize radicals along the polymer backbone.^27^ In the case of PCTFE, after 20 h, a decrease in carbon-centered radicals (*g*-factor ≈ 2.004) was observed, followed by an increase in other radical species, potentially peroxyl radicals (*g*-factor ≈ 2.011), as previously mentioned, which resulted in a significant increase in the absolute area under the peak. This could be a possible explanation for the increase in radicals observed in PCTFE over time. We hypothesize that for this specific substrate (PCTFE), oxygen was trapped within the amorphous regions of the material during the manufacturing process. During the irradiation, the trapped oxygen molecules can react with available carbon-centered radicals to form peroxyl radicals. Peroxyl radicals are known to propagate the oxidation and degradation reactions within the substrate, thus increasing the number of radicals observed over time.^28^

**Table 2:** Characteristics of the EPR Signal(s) Produced in Fluoropolymer Substrates Following Ionizing Radiation; % Change of Radicals was Determined after *t* ≥ 20 h

	EPR signal	
substrate	*g*-factor	% change^a^
FEP	2.018	–10%
PCTFE	2.011, 2.004	+102%
PVF	2.005	–39%

a Determined using the change in doubly integrated areas of continuous wave EPR spectra.

### 3.3 SEM/EDS Analysis of PEM Grafting Uniformity

SEM/EDS analysis of the synthesized PEMs was conducted to determine the grafting profile through the membrane cross-section. If grafting is highly successful, the carbon to fluorine atomic ratio should increase in the membrane and stay relatively constant across the cross-sectional area of the membranes. Changes in electron density are also expected because the fluoride groups have a higher electron density compared to hydrogen. These electron density differences mean that the grafting concentration gradient can be observed in SEM images. PEMs were embedded in epoxy and cross-sectioned using a microtome. EDS analysis was used to track the carbon to fluorine ratio through the membrane to determine the relative concentration of the grafted monomer.

The SEM image in Figure 4(B) shows the cross-section of a developed PEM. This sample was synthesized using indirect radiation grafting of 4-vinylpyridine onto a FEP substrate (sample IV in Table 1). The sample had approximately 18.6% by weight grafting. The EDS spectrum collected at the center of the cross-section (green line shown in Figure 4) showed an atomic composition by mass of C—71%, N—3%, O—9%, and F— 15%. The ratio of carbon to fluoride after 4 measurements on different cross-sectional areas of the sample is 4.8 ± 0.6, which is substantially higher than the initial FEP substrate ratio of 0.6 ± 0.2 measured for ungrafted FEP. The increase in the carbon to fluorine ratio shows that monomer grafting was achieved through the center of the membrane (bulk grafting).

**Figure 4:**
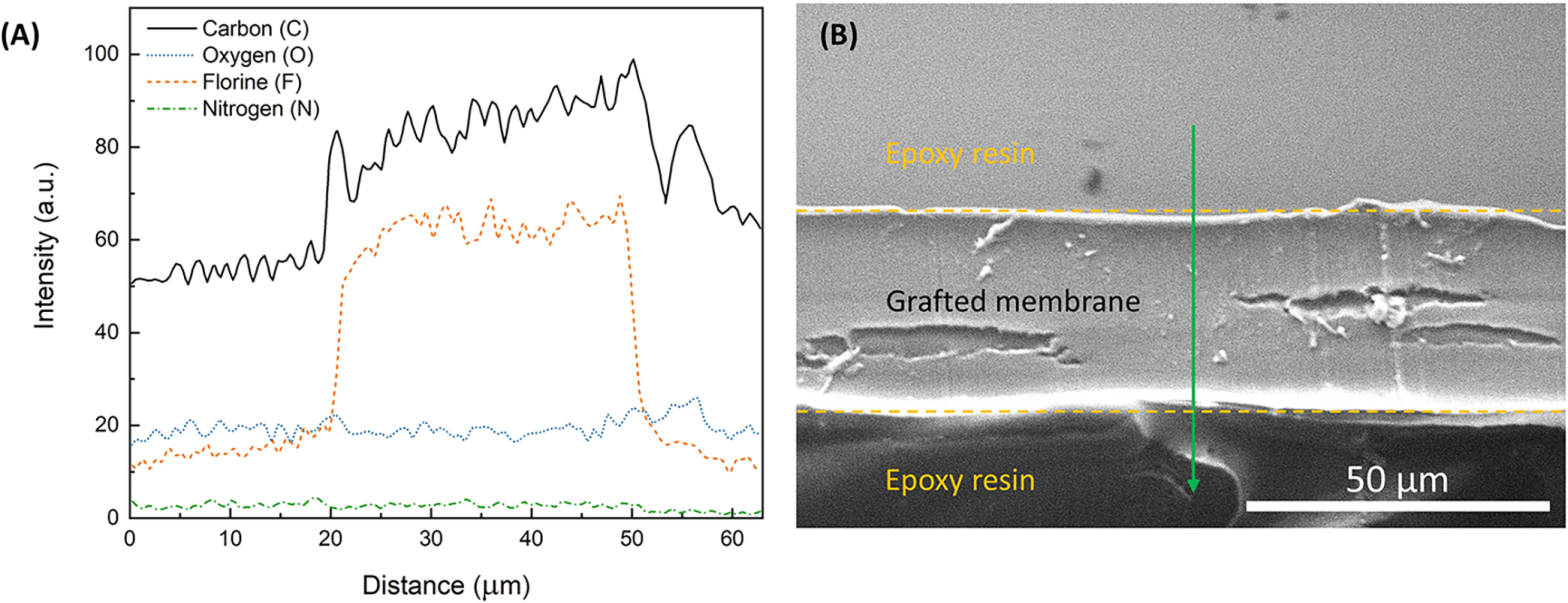
SEM/EDS results of green line scan test area (A) and SEM image (B) of the cross-section of PEM membrane synthesized using indirect radiation grafting for 4-vinylpyridine onto FEP substrate.

The grafting profile through the membrane was determined by EDS line scans of the cross sections of the samples. Representative data of these line scans for FEP are shown in Figure 4(A). The region of this particular SEM/EDS line scan on the cross-sectional area of the sample is shown by the green arrow in Figure 4(B). The results of the EDS line scan show the gradient through the membrane with a steady concentration of fluorine (orange) across the cross-section of the FEP film. This indicates that the grafting is uniform through the membrane. Some changes in the atomic composition of the line scans can be attributed to the roughness of the sample surface. These measurements were conducted for all of the developed fluorocarbon PEMs in this study, synthesized using a high-dose rate of 1000 kGy/h. Similar to the FEP data presented in Figure 4, all fluorocarbon PEMs revealed uniform cross-sectional atomic compositions.

### 3.4 FTIR Analysis of PEMs Prior to and Post Acid Treatment

Chemical composition analysis, via ATR-FTIR, was performed to identify chemical changes within the synthesized PEM by comparing the FTIR spectrum before and after radiation grafting of the different heterocyclic amine monomers into the substrates, confirming that the materials were successfully grafted. This ATR technique is primarily investigating the surface of the materials synthesized (depth penetration of nominally 0.5 to 4 μm) and does not provide information about the bulk properties and chemistry. The same analytical technique was used to better understand the effects of the acid treatment on the prepared PEMs. The acid treatment process was performed to simulate fuel cell conditions and protonate the PEM for electrochemical impedance spectroscopy measurements.

The absorption spectra presented in Figure 5 are one representative example of the materials studied in this work. More specifically, in Figure 5(A), we show the full spectra of PVF substrate (black), grafted 4-vinylpyridine on PVF PEM (red), 5% nitric acid-treated PVF PEM (green), 5% sulfuric acid PVF PEM (blue), and 5% phosphoric acid PVF PEM (aqua). The data indicate that after radiation grafting, as shown in Figure 5(B), there are two medium intensity peaks for 4-vinylpyridine at 1598 and 1412 cm^–1^ associated with C=C and C=N stretching within the aromatic pyridine group and C–H bending vibration (scissoring mode of CH_2_), respectively. Also, a smaller intensity peak at 1217 cm^–1^ indicated the presence of C–N bonds (stretching mode). Finally, a peak at 818 cm^–1^ confirms the presence of C–H bonds representing the out-of-plane bending vibration of C–H in the aromatic ring. These peaks indicate successful grafting of the heterocyclic amine monomer into the substrate.^29^ Furthermore, there is no absorption of water observed in the region around 3300 cm^–1^.

**Figure 5:**
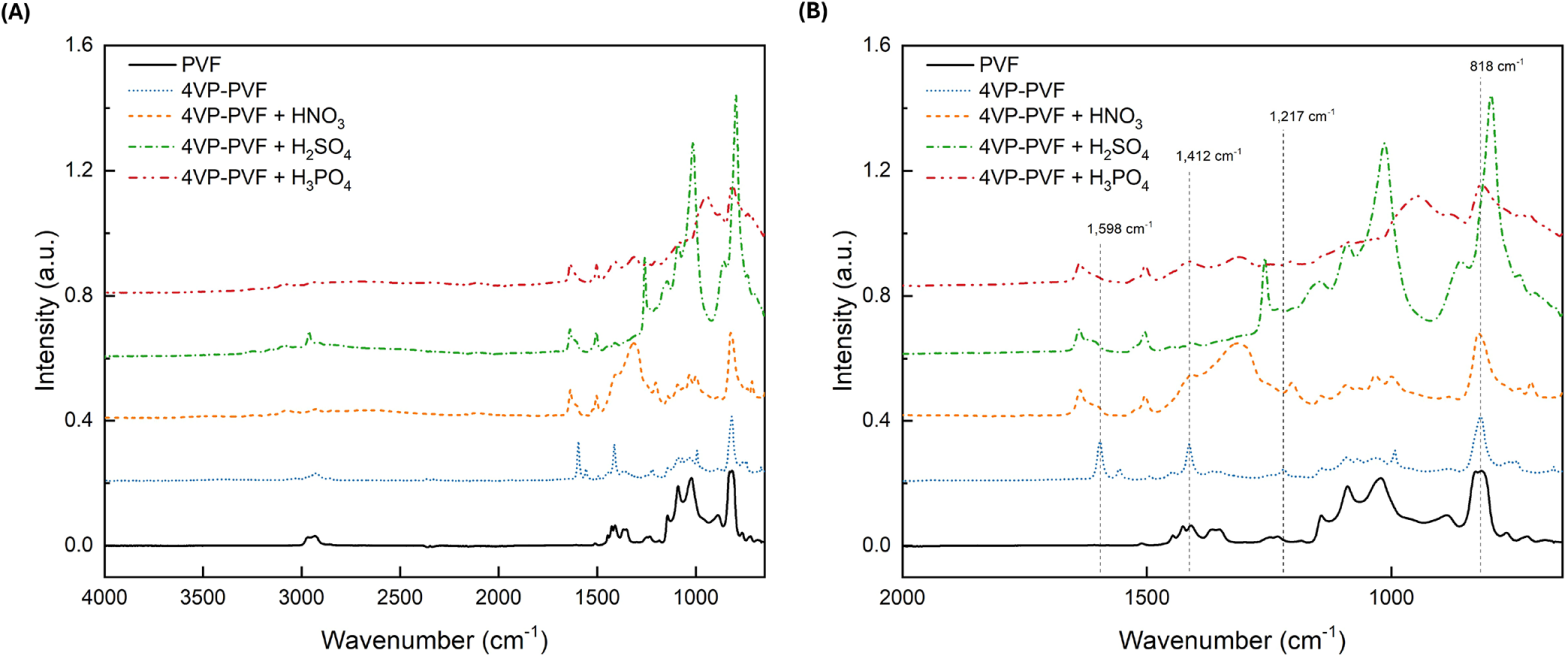
FTIR on PEMs synthesized with 4-vinylpyridine grafted on PVF; (A) full spectrum data shown for substrate, grafted PEM, and the materials after various acid treatments, and (B) spectra focused on the wavenumber area of interest for the synthesized material.

Figure 5 also shows the impact of acid treatments of nitric, sulfuric, and phosphoric acids on the developed PEMs. The acid treatment with the aforementioned acids caused the 4-vinylpyridine peaks to shift to the left, which indicates the protonation of the pyridine group.^29^ Some additional medium and weak intensity peaks also appear on the FTIR spectra after the acid treatment step due to the conjugate acids. These results show that the acid treatment used to protonate the PEMs, before the EIS measurements, will not alter the chemical composition of the membranes.

### 3.5 PEM Conductivity

The electrochemical performance of PEMs synthesized with 5-vinylpyrimidine on PVF (sample III in Table 1) is summarized in Figures 6 and 7. These membranes were selected for their high average grafting values relative to the other PEM formulations and their superior performance during the proton conductivity evaluation at anhydrous conditions (as shown later in Figure 8). The control of two experimental variables, relative humidity (R.H.) and temperature, allows us to compare the proton conductivity of these membranes and provide a comparison to a benchmark sample of Nafion membranes (provided by 3M), under fully hydrated conditions and as the membranes are dehydrated. Previous work with Nafion membranes suggests that DRT calculated relaxation times for proton conductivity through the PEMs occur between 10^–4^ s and 10^–2^ s.^30,31^ The N–H hydrogen bond channels in heterocyclic amine PEMs are expected to behave similarly to the hydrogen bond system found within the Nafion membranes, and by using DRT, we can distinguish between electrode and membrane reactions to determine the proton conductivity of the membrane.

**Figure 6:**
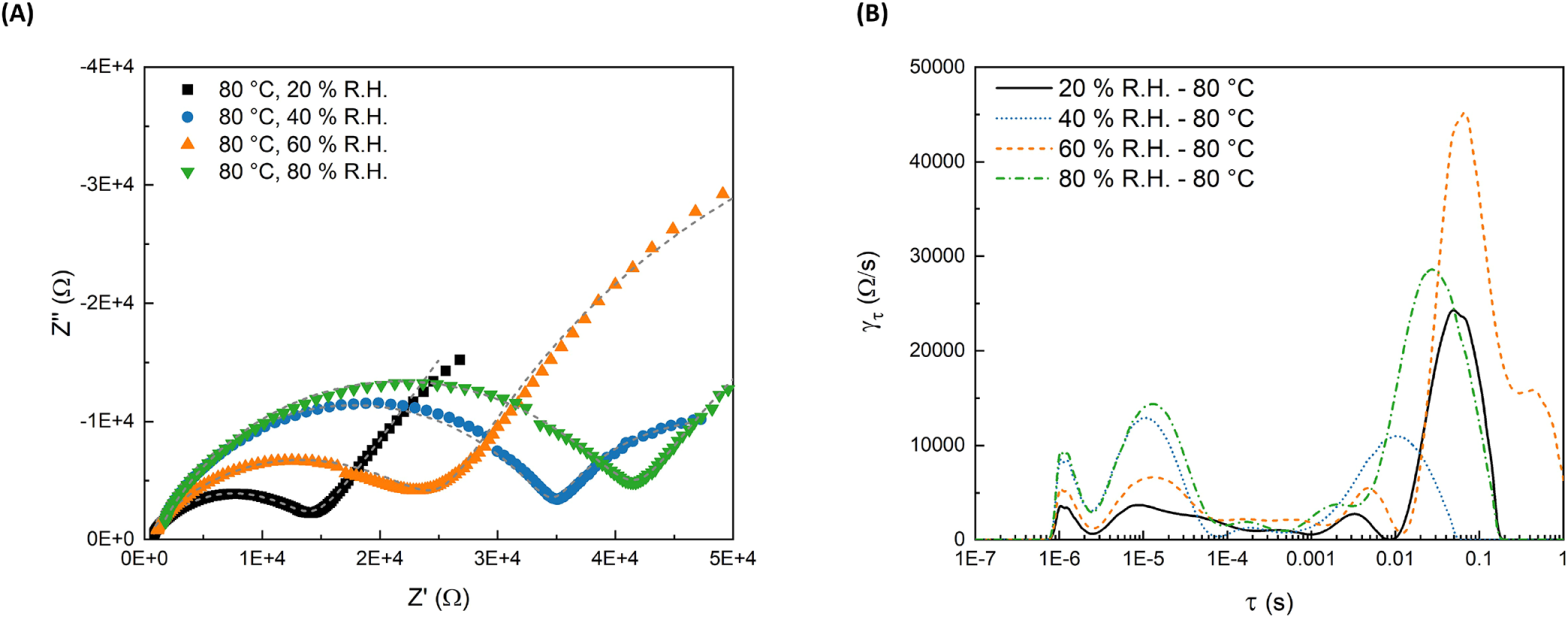
Analysis of PVF–PEMs data as a function of humidity using a Pt 4-point probe; (A) Nyquist Plot of EIS data; and (B) DRT analysis.

**Figure 7:**
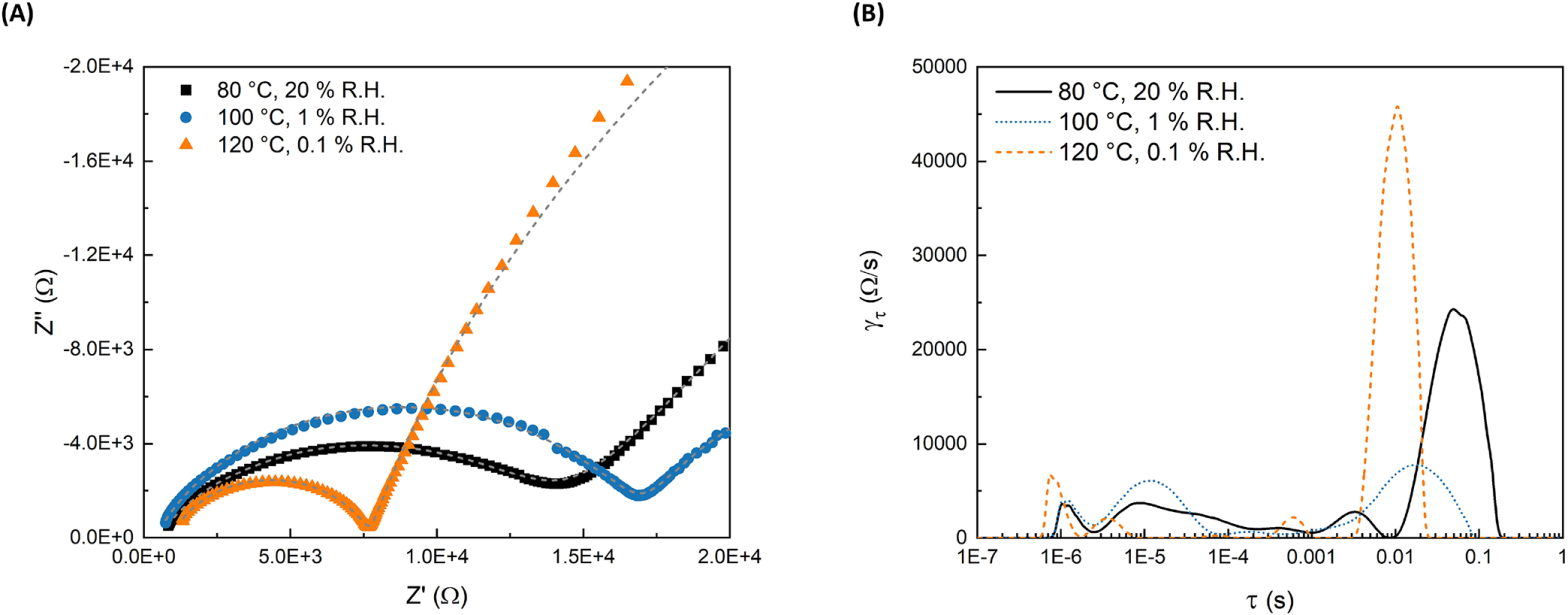
Analysis of PVF–PEMs data as a function of temperature using a Pt 4-point probe; (A) Nyquist Plot of EIS data; and (B) DRT analysis.

**Figure 8:**
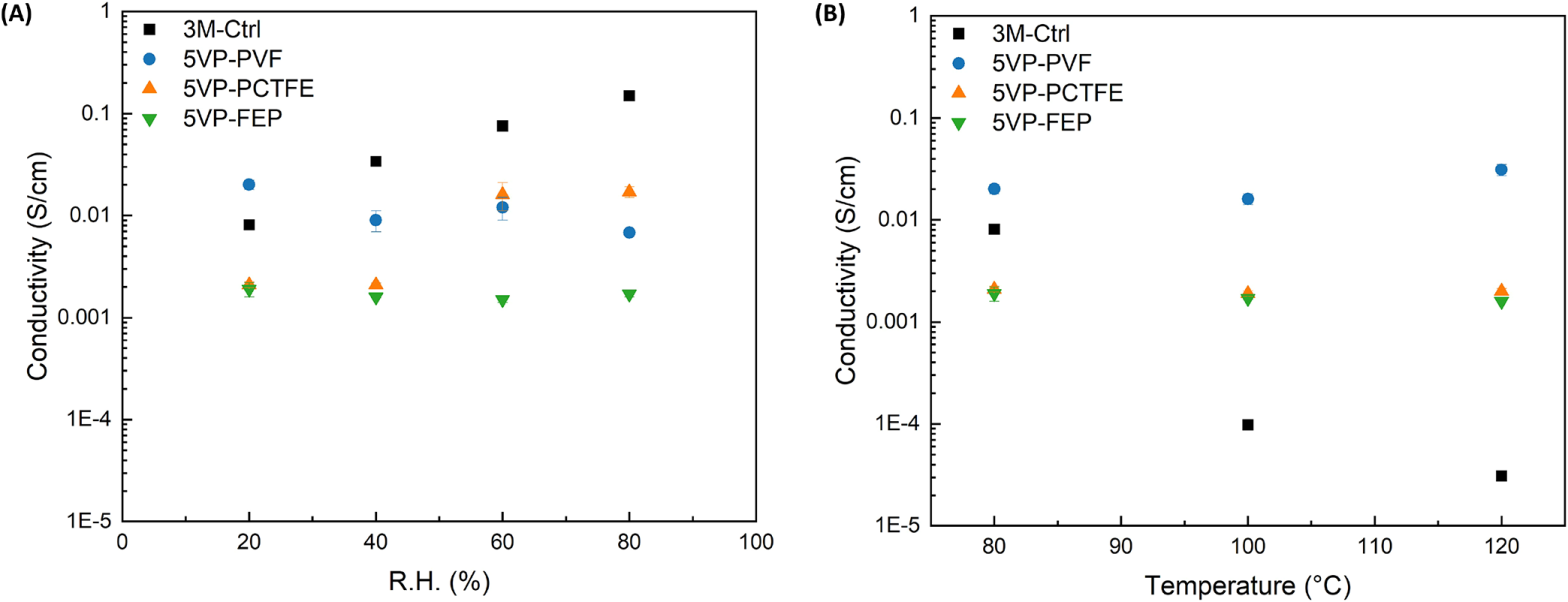
EIS proton conductivity of 5% HNO_3_-treated PEMs using a Pt 4-point probe; (A) as a function of relative humidity at 80 °C, and (B) as a function of temperature under dehydrating conditions. A 3 M control sample was also measured and compared to the synthesized PEMs in this study. Data presented are mean ± standard error of the mean (S.E.M.), with *n* ≥ 3.

Impedance spectra for 5-vinylpyrimidine (5VP) on PVF (sample III in Table 1) at 80 °C as the humidity was raised from 20% R.H. to 80% R.H. are shown in Figure 6(A), and the best fit results using the equivalent circuit are shown as dashed lines. There was no significant trend between the humidity and proton conductivity in the PVF–PEMs. DRT in Figure 6(B) shows a stable time constant between 10^–3^ s and 10^–1^ s, suggesting stable water-driven proton transport.^30,32^ The stability in peak location with humidity shows that the electrochemical reactions involved remained the same. The slight shift in peak positions suggests that the hydrated channels may change slightly, but the shift is likely not significant enough to indicate a new dominant transport mechanism in the membrane. These results informed the R­(RC)­(RC) equivalent circuit model used to extract the proton transport impedance values for further comparison.

More importantly, to enable higher temperature PEMFC operation, Figure 7 explores the effect of temperature with decreasing humidity. As the temperature increases to 120 °C, in Figure 7(B), there is a new small DRT proton conductivity peak formed at 0.6 ms, opposite to what was observed with the 3 M control (Supporting Figures S1 and S2). Also, at the highest temperature of 120 °C, well beyond water’s boiling point, the distribution function of relaxation times γ_τ_ shifts toward shorter time constants. This trajectory suggests that the proton-conductive pathway changes from a water-filled conductive network to a mechanism where the protons hop across hydrogen-bonded monomer functional groups grafted to the PEM. The change in proton conductivity mechanism was reflected by the positive trend in proton conductivity observed under dehydrating conditions, as shown in Figure 8. Under dehydrating conditions, water could no longer be used as the medium for proton conductivity and ionic groups are forced closer together, supporting proton hopping between functional groups. The temperature-dependent data were fit to the same model as the R.H. data, with only the interpretation of the mechanism for proton conductivity changing at the highest temperatures.

With DRT-identified relaxation constants, we confirmed that these radiation-grafted membranes display similar conductive properties to Nafion membranes (see Supporting Figure S1c,d), which allows us to extract the proton conductivity unambiguously from equivalent circuit modeling. In this case, we are only concerned with *R*_PEM_, the resistance of the membrane to proton transport. The fitted results from Figures 6 and 7 were converted to conductivity for both the humidity-controlled experiment and the temperature-controlled experiment, as shown in Figure 8(A,B), respectively.

Figure 8(A) compares three radiation-grafted PEM materials with a 3 M control. The 3 M control material (black) shows an increase in conductivity with increased humidity, indicating that the water-filled conductive network swells with humidity. FEP (sample I in Table 1) and PVF (sample III in Table 1) show little to no humidity dependence in their conductive properties. The difference in conductivity is likely due to the difference in the grafting density. Unlike the other radiation-grafted membranes, PCTFE (sample II in Table 1) displayed a stepwise conductivity increase between 40% R.H. and 60% R.H. This result suggests that the water uptake of the PCTFE membrane spontaneously causes swelling when the humidity reaches a critical value. Further research is necessary to determine whether this is a property of the support material, the relatively low grafting density, or both.

While humidity-dependent data are important to establish a mechanistic comparison to sulfonic acid-based membranes, the conductivity under dehydrating conditions and high temperatures is critical to enable higher temperature PEMFC operation. Figure 8(B) shows the conductivity of these membranes derived from the data presented in Figure 7. The 3 M control (black) represents a traditional fuel cell membrane that uses water channels for proton conductivity, which is confirmed in Figure 8. Without an aqueous support system, the proton conductivity of the 3 M control membrane decreased by nearly 3 orders of magnitude as the temperature approached and exceeded 100 °C. Although the radiation-grafted membranes may have water-filled channels under high humidity conditions, they also have an anhydrous conduction pathway through proton hopping between the grafted functional groups, and subsequently they maintain consistent conductivity as the temperature exceeds 100 °C. Functionalized PVF PEMs had the highest proton conductivity because the PVF substrate supported a higher degree of grafting and affinity for the selected heterocyclic amine monomers. A stable and relatively tight monomer network is required for proton hopping between functional groups. Refining the structure of proton-conductive channels in these PEMFCs can further improve the proton conductivity for high-temperature applications.

### 3.6 Small-Angle Neutron Scattering

Small-Angle Neutron Scattering (SANS) measurements were conducted to determine the nanostructure of the grafted proton-conductive monomers and fluorocarbon substrate. To illustrate the structural details that lead to the material properties examined above, we chose to measure the scattering of our best-performing anhydrous proton conductor, PVF with 5-vinylpyrimidine (sample III in Table 1), grafted to it. Figure 9 shows the evolution of the SANS signal as untreated PVF is modified and subsequently hydrated with water and acid. Immediately apparent in each data set is that the intensity plotted against the scattering vector shows power law behavior at low *q* values (i.e., *q* < 0.1 nm^–1^). At larger *q* values (i.e., between 0.2 nm^–1^ and 0.6 nm^–1^), a peak emerges. To assess these features quantitatively, we fit a combined power law, the Gaussian peak model (eq 2), to the scattering data

2 $$I_{q}=I_{p}q^{-m}+I_{G}\exp\left(-\frac{1}{2}\frac{1\left(q-q_{0}\right)}{\sigma }\right)+I_{B}$$

**Figure 9:**
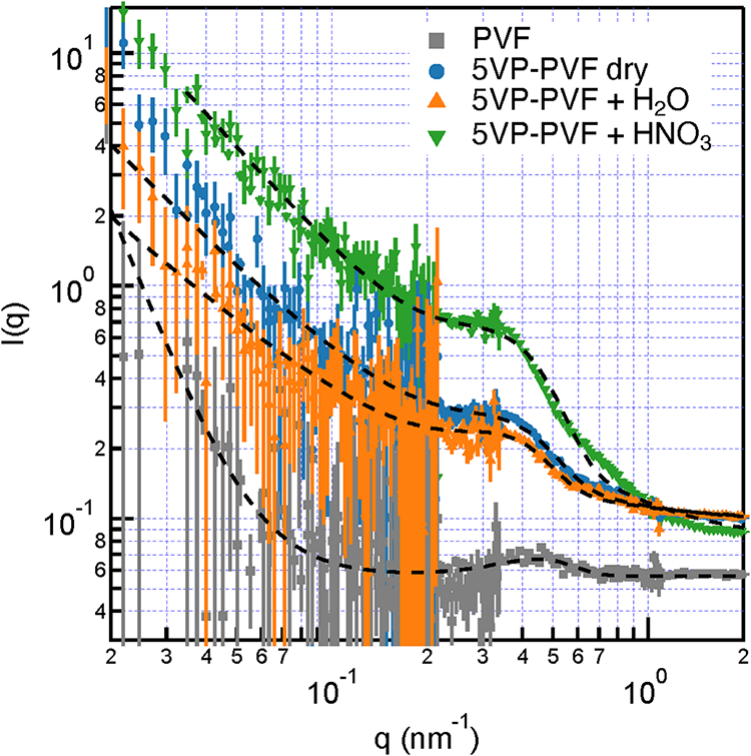
SANS measurements reveal structural changes of the membrane and ionomer leading to a functional proton transport membrane: PVF membrane (gray), 5VP-PVF in air (blue), 5VP-PVF treated with H_2_O (orange), 5VP-PVF treated with HNO_3_ (green). Error bars represent 1 SD, and dashed lines are best fits of eq 2 to the data.

where *I_q_* is the scattering intensity, *m* is the power law exponent, *q*_0_ is the peak position, σ is the peak standard deviation, *I*_p_ and *I*_G_ are scaling factors for the power law and Gaussian peak models, respectively, and *I*_B_ is the incoherent background. Results are summarized in Figure 9 and Table 3. Measurement conditions limit our ability to draw significant conclusions from the low *q* data; however, the fitting suggests that unmodified PVF presents a three-dimensional scattering cross-section based on the *m* ≈ 3.5 power law dependence, although the noise in the data precludes any definitive analysis. When 5-vinylpyrimidine is grafted to the PVF, the low *q* data shift to a lower dimensionality, below 1.5, suggesting that there is a persistent 1-to-2-dimensional scattering interface. More critical for proton conduction, the Gaussian peak observed in the intermediate *q* region suggests that there is a significant scattering component that shifts from ≈0.44 nm^–1^ for PVF to ≈0.34 nm^–1^ for 5VP-PVF. This feature is often attributed to the presence of an ionomer in ionic conducting membranes.^33^ These values suggest that PVF has an embedded scattering object with a 14 nm repeat structure that expands to ≈18 nm when pyrimidine is grafted to it. This is stable under neutral pH hydration conditions but swells slightly to ≈19 nm in the presence of nitric acid.

**Table 3:** Structure-Dependent Parameters for Combined Power Law-Gaussian Peak from SANS Data on a Grafted PVF Substrate^a^

	*m*	*q*_0_ (nm^‑1^)	σ (nm^‑1^)	χ^2^
PVF	3.5 ± 0.2	0.443 ± 0.008	0.124 ± 0.008	1.68
5VP-PVF	1.374 ± 0.008	0.340 ± 0.003	0.137 ± 0.004	3.37
5VP-PVF (hydrated)	1.22 ± 0.01	0.343 ± 0.003	0.122 ± 0.003	2.37
5VP-PVF(acid-treated)	1.315 ± 0.005	0.325 ± 0.001	0.100 ± 0.001	2.10

a Reported uncertainties are for individual fits using the 95% confidence interval from 9000 samples post convergence.

## 4 Conclusions

The goal of this research was to design, synthesize, and analyze solid-state PEMs that incorporate proton exchange centers and to assess them for high-temperature conductivity for fuel cell applications. PEMs were successfully synthesized by using indirect radiation grafting to combine the material properties of protic heterocyclic monomers with fluorocarbon-polymeric substrates. The symmetrical structures of 4-vinylpyridine and 5-vinylpyrimidine, once grafted onto the polymer substrates in the desired amounts, give these PEMs a favorable microstructure for anhydrous proton transport. The fluorocarbon-polymeric substrates FEP, PCTFE, and PVF were selected for their radiation processing characteristics, including high radiation resistance and low backbone scission byproducts. Indirect grafting was required to avoid homopolymerization reactions at the membrane surface and homopolymer production from vinyl-activated cross-linking. This approach covalently grafted the vinyl group of the ionic liquid monomers into amorphous regions of the fluorocarbon substrates. The rate of free radical decay in the fluoropolymers correlated with the degree of fluorination (FEP > PCTFE > PVF), potentially indicating that fluoride substitution helps prevent radicals from decaying through cross-linking reactions, by immobilizing them along the polymer backbone, thereby preserving them as grafting sites.

The PEM test results established that the proton transport of 5VP produced temperature-independent conductivity on all polymer substrates. While the grafting extended to the center of the membrane, grafting densities >100% suggest that 5VP conductivity networks are not limited to monomer-to-monomer proton transport. SEM and SANS analysis suggest that this network is uniform throughout the film without introducing any deleterious swelling. This simple preparation workflow suggests that indirect radiation grafting is a promising route for the fabrication of high-quality PEMs that can be optimized to operate under the high thermal and humidity demands of a hydrogen fuel cell.

## Supplementary Material



## Footnotes

1 Certain commercial materials, equipment, and instruments may be identified in this work to describe the experiments as completely as possible. In no case does such an identification imply a recommendation or endorsement by the National Institute of Standards and Technology, nor does it imply that the materials, equipment, or instrument identified are necessarily the best available for the purpose.

## Notes

Additional experimental details and electrochemical impedance spectroscopy (EIS) data for 3 M control PEMs, including graphs for EIS and DRT analysis (PDF)
