# Supplementary material for: Radiation-Grafted Polymer Electrolyte Membranes for Anhydrous Fuel Cell Operation
Source: ACS Polym Au. 2026 Jan 21;6(1):293–304. doi: 10.1021/acspolymersau.5c00142 (PMC12903426; doi:10.1021/acspolymersau.5c00142)
Supplement: Supplementary file 1 [file lg5c00142_si_001.pdf]

Supplemental Information

Radiation Grafted Polymer Electrolyte Membranes for Anhydrous Fuel Cell Operation

Kevin R. Mecadon[#, a], Zois Tsinas[#, b, c], Joseph W.F. Robertson[d], Markus Bleuel[e], Eric D. Wachsman[a], Fred B. Bateman[f], and Mohamad I. Al-Sheikhly[*, a]

[a] *Department of Materials Science and Engineering, University of Maryland, College Park, MD 20742, USA*
[b] *Theiss Research, La Jolla, CA 92037, USA*
[c] *Materials Measurement Science Division, National Institute of Standards and Technology, Gaithersburg, MD 20899, USA*
[d] *Microsystems and Nanotechnology Division, National Institute of Standards and Technology, Gaithersburg, MD 20899, USA*
[e] *NIST Center for Neutron Research, National Institute of Standards and Technology, Gaithersburg, MD 20899, USA*
[f] *Radiation Physics Division, National Institute of Standards and Technology, Gaithersburg, MD 20899, USA*
[#] These authors contributed equally to this work
[*] Corresponding author
Email address: mohamad@umd.edu

Electrochemical Impedance Spectroscopy 1

References..3

Electrochemical Impedance Spectroscopy

Figure SI_1 shows the electrochemical impedance spectroscopy (EIS) and distribution of relaxation time (DRT) analysis results for the 3M 825EW control PEM under variable temperature and humidity conditions. The Nyquist plot of EIS data of 3M 825EW control and the DRT analysis as a function of humidity at 80 °C is shown in Figure SI_1(A) and (C), respectively. As humidity decreases, the impedance of the PEM membrane increases due to dehydration of the membrane. Figure SI_1(B) and (D) shows the Nyquist plot of EIS data of 3M 825EW control and DRT analysis as a function of temperature. The number of peaks in the DRT spectrum supports the electrochemical model that was used to analyze the EIS data. Figure SI_1 (C) shows that as relative humidity decreases, at 80 °C, there is an increase of the peak at 0.5 s (2 Hz) which we attribute to low current due to a reduced mass transport of protons at the electrode. Under these conditions, water is the primary method for proton conductivity and charge transport thermodynamics [1]. Once the membrane drops to 20 % R.H. at 80 °C, the mechanism for conductivity changes, as shown by the emergence of a second DRT peak at 0.002 s (500 Hz, black). This frequency range correlates to the hydrogen bond relaxation time within the nanochannels used for proton conductivity and this peak shift was observed in literature for Nafion [2, 3]. Figure SI_1(D) shows a shift in DRT peaks observed at 100 °C in which the membranes continue to dehydrate, changing the medium for proton conductivity from water to the functional groups in the membranes. This

mechanism shift in DRT peak location was also observed in DRT analysis of Nafion membranes tested under dry hydrogen conditions by M. A. Travassos et al. [4].

PEMs similar to Nafion that rely on water for proton conductivity usually show strong humidity dependence since hydration allows the proton conductive nanochannels in the amorphous region to function [2]. The 3M 825EW control membranes show four orders of magnitude decrease in proton conductivity due to dehydration as the humidity decreases. As the temperature is increased above 100 °C (and the R.H. decreases by a factor of ten), there are two additional peaks in the DRT in the 0.01 s (100 Hz) to 1s (1 Hz) region. These peaks likely represent the hydrogen bond network becoming more mobile with increased lattice vibrations at this temperature. This results in improved localized mobility of protons and limited diffusion to the electrodes [3]. The 3M sample's change in relaxation time with humidity is an example of a shift in proton conductive mechanisms in the membrane.

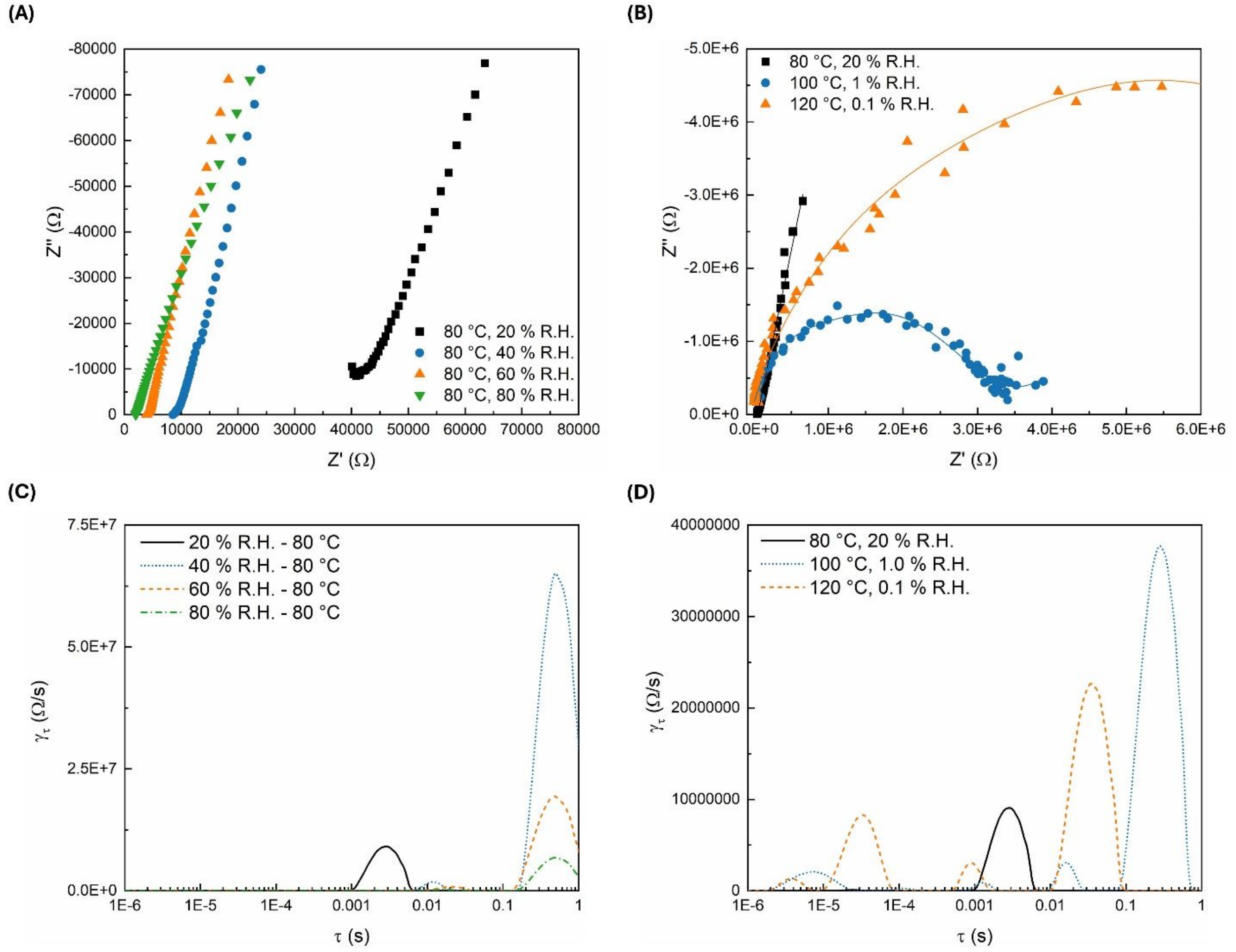

Figure SI_1. Analysis of Pt 4-point probe EIS data 3M 825EW control; Nyquist Plot of EIS data (A) function of humidity, (B) function of temperature and humidity combined (including fits), (C) DRT analysis at 80 °C and 20 %, 40 %, 60 % and 80 % relative humidity, and (D) DRT analysis of combined temperature and humidity dependance.

References

[1] P. Choi, N.H. Jalani, R. Datta, Thermodynamics and proton transport in nafion: II. Proton diffusion mechanisms and conductivity, Journal of the electrochemical society 152(3) (2005) E123.

[2] H.E. Andrada, M.B. Franzoni, A.C. Carreras, F.V. Chávez, Dynamics and spatial distribution of water in Nafion 117 membrane investigated by NMR spin-spin relaxation, international journal of hydrogen energy 43(18) (2018) 8936-8943.

[3] S.J. Osborn, M.K. Hassan, G.M. Divoux, D.W. Rhoades, K.A. Mauritz, R.B. Moore, Glass transition temperature of perfluorosulfonic acid ionomers, Macromolecules 40(10) (2007) 3886-3890.

[4] M.A. Travassos, V.V. Lopes, R. Silva, A.Q. Novais, C. Rangel, Assessing cell polarity reversal degradation phenomena in PEM fuel cells by electrochemical impedance spectroscopy, International journal of hydrogen energy 38(18) (2013) 7684-7696.
